# Proteasome-associated deubiquitinase ubiquitin-specific protease 14 regulates prostate cancer proliferation by deubiquitinating and stabilizing androgen receptor

**DOI:** 10.1038/cddis.2016.477

**Published:** 2017-02-02

**Authors:** Yuning Liao, Ningning Liu, Xianliang Hua, Jianyu Cai, Xiaohong Xia, Xuejun Wang, Hongbiao Huang, Jinbao Liu

**Affiliations:** 1 State Key Lab of Respiratory Disease, Protein Modification and Degradation Lab, Department of Pathophysiology, Guangzhou Medical University, Guangdong, China; 2 Guangzhou Institute of Cardiovascular Disease, The Second Affiliated Hospital, Guangzhou Medical University, Guangzhou, Guangdong, China; 3 Division of Basic Biomedical Sciences, Sanford School of Medicine of the University of South Dakota, Vermillion, USA

## Abstract

Androgen receptor (AR) is frequently over-expressed and plays a critical role in the growth and progression of human prostate cancer. The therapy attempting to target AR signalling was established in decades ago but the treatment of prostate cancer is far from being satisfactory. The assignable cause is that our understanding of the mechanism of AR regulation and re-activation remains incomplete. Increasing evidence suggests that deubiquitinases are involved in the regulation of cancer development and progression but the specific underlying mechanism often is not elucidated. In the current study, we have identified ubiquitin-specific protease 14 (USP14) as a novel regulator of AR, inhibiting the degradation of AR via deubiquitinating this oncoprotein in the androgen-responsive prostate cancer cells. We found that (i) USP14 could bind to AR, and additionally, both genetic and pharmacological inhibition of USP14 accelerated the ubiquitination and degradation of AR; (ii) downregulation or inhibition of USP14 suppressed cell proliferation and colony formation of LNcap cells and, conversely, overexpression of USP14 promoted the proliferation; and (iii) reduction or inhibition of USP14 induced G0/G1 phase arrest in LNcap prostate cancer cells. Hence, we conclude that USP14 promotes prostate cancer progression likely through stabilization of AR, suggesting that USP14 could be a promising therapeutic target for prostate cancer.

Androgen receptor (AR) signalling pathway dominates the survival, proliferation and growth of prostate cancer. AR is a ligand-dependent transcription factor, belonging to the nuclear receptor superfamily.^1, 2, 3^ In cytoplasm, androgen ligands, such as dihydrotestosterone, directly bind AR, which induces rapid phosphorylation of the AR and its translocation into the nucleus. Subsequently, the ligand-activated AR binds to specific DNA sequences on the target genes and initiates expression of a series of genes that promote prostate cancer progression. For instance, kallikrein-related peptidase 3 (also known as prostate-specific antigen, PSA) is the best characterized AR target, which is used in the clinic to monitor prostate cancer development and progression.^4, 5^ In addition, AR gene amplification and mutation are associated with prostate cancer development and the progression from androgen-dependent prostate cancer to castrate-resistant prostate cancer, which renders the cancer incurable.^6, 7, 8^

Given the pivotal role of AR signalling in prostate cancer development, AR-based therapy was born several decades ago. However, the current anti-prostate cancer strategies in the clinic cannot fully cure the disease. Recent studies have attached importance to the dysregulated AR expression in prostate cancer and its underlying mechanisms because these represent the most therapeutically relevant targets in this disease.^2^ Although numerous researches have focused on the regulation of AR synthesis in prostate cancer, the regulation of AR post-translational modification and degradation has been historically underappreciated. Nevertheless, it has been demonstrated that Akt and E3 ligase MDM2 form a complex with AR and promote phosphorylation-dependent AR ubiquitination, resulting in AR degradation by the proteasome.^9^ This implies that AR-centred signalling could potentially be regulated by altering AR protein stability, which prompted us to examine the potential role of deubiquitinases (DUBs) in the regulation of AR protein degradation.

In the ubiquitin proteasome system, ubiquitination and deubiquitination are two reversible events that counter one another and control the stability of most cellular proteins. Specifically, protein ubiquitination transforms the function and location of the target protein or promotes its degradation, while this process can be reversed by deubiquitination. In eukaryotic organisms, DUBs remove ubiquitin (Ub) and ubiquitin-like (Ubl) chains from target proteins prior to their degradation and thereby participate in the regulation of multiple cellular processes, including cell cycle control,^10, 11^ DNA stabilization,^12, 13, 14^ chromatin modification^15^ and various cellular signalling pathways.^16^ The human genome encodes approximately 100 putative DUBs, which are subdivided into six families on the basis of their sequences and structural differences. Recently, several DUBs are reported to be associated with the co-regulation, stabilization or transcription of AR. For example, USP26 physically interacts with AR and influences AR ubiquitination and transcriptional activation;^17^ USP12 stabilizes AR, enhances its cellular function, and thereby triggers the gene expression of PSA.^18^ In addition, USP10 also has been reported to bind AR, resulting in increased transcriptional activity. Overexpression of wild-type USP10 stimulated AR activity as revealed by reporter constructs harbouring selective androgen response elements, non-selective steroid response elements or the mouse mammary tumour virus promoter. USP10 reduction impaired the mouse mammary tumour virus response to androgen.^19, 20^ Moreover, USP7 seems to be required for binding of the AR to chromatin and mediates its activity. USP7 was detected in the AR-containing protein complex assembled on the androgen response elements of *FKBP5*, *PSA* and *PDE9A* upon dihydrotestosterone stimulation, which are required for the proliferation of prostate cancer cells.^21^

To date, the regulation of AR by DUBs associated with the 19S regulatory particle of the proteasome complex remains poorly understood. There are three identified DUBs associated with the proteasome: USP14, UCHL5 and Rpn11 in mammalian cells. Rpn11 is a stoichiometric subunit of the lid subcomplex of the 19S regulatory particle whereas USP14 and UCHL5 reversibly associate with the 19S, indicative of attractive and versatile roles for these molecules.^22, 23, 24, 25^ As a member of the ubiquitin-specific processing protease family, USP14 has been reported to be highly expressed in several kinds of carcinoma, including multiple myeloma,^23^ ovarian carcinoma^26^ and colorectal cancer.^27^ In this study, we have identified that USP14 promotes the cell cycle in prostate carcinoma cells by deubiquitination and stabilization of AR.

## Results

### USP14 positively regulates the proliferation of androgen-responsive prostate cancer cells

**Figure 1:**
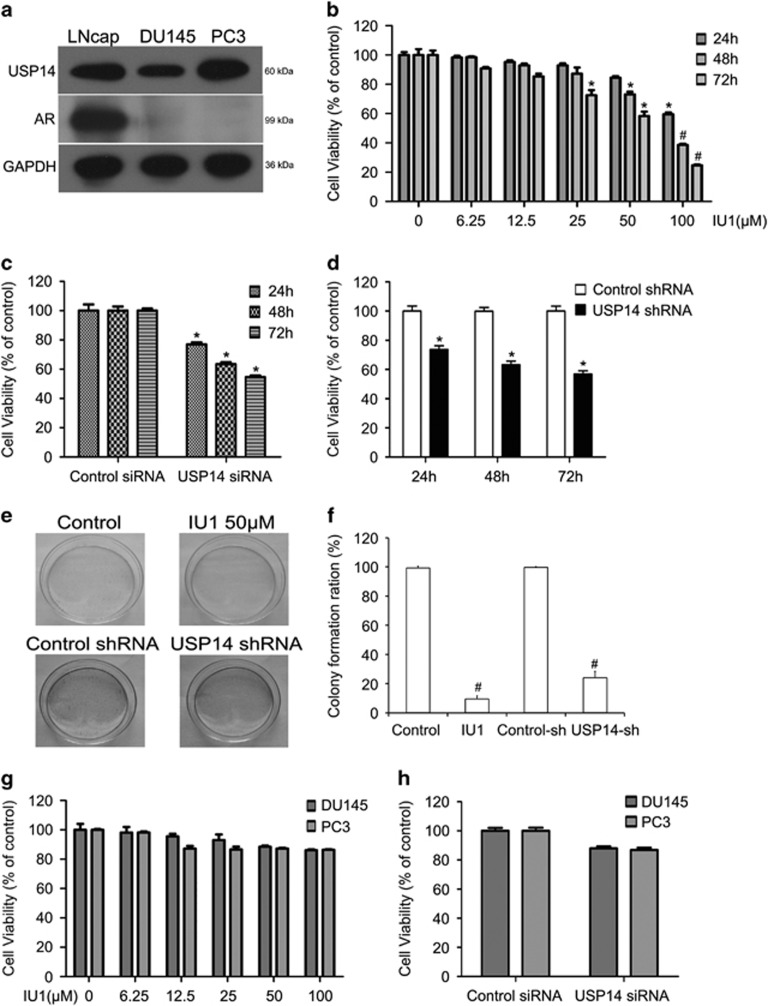
USP14 inhibition or silence reduced cell proliferation in androgen-responsive cancer cells. (**a**) Total proteins were extracted from the cultured cells and subjected to western blot analysis using antibodies against USP14 and AR. GAPDH was used as a loading control. (**b**) LNcap cells were treated with the indicated concentrations of IU1 for 24, 48 and 72 h. (**c**) LNcap cells were treated with siRNA for 24, 48 and 72 h. (**d**) LNcap cells were treated with shRNA for 24, 48 and 72 h. Cell viability was detected by MTS assay. Error bars correspond to 95% confidence intervals of three independent experiments. *P<0.05, ^#^*P*<0.01 using two-sided *t*-test. (**e**) LNcap cells exposed to IU1 50 *μ*M or shRNA 48 h were suspended in 30% agarose for 2 weeks, representative images were shown, and (**f)** the numbers of colonies were counted. Error bars correspond to 95% confidence intervals. ^#^*P*<0.01, compared with control treatments. (**g**) DU145 or PC3 cells were treated with the indicated concentrations of IU1 for 48 h. (**h**) DU145 or PC3 cells were treated with siRNA for 48 h. Cell viability was detected with MTS assay. Error bars correspond to 95% confidence intervals of three independent experiments

First we observed that USP14 was expressed in both androgen-responsive prostate cancer LNcap cells and androgen-irresponsive prostate cancer DU145 and PC3 cells. We also verified that AR was highly expressed in the LNcap cells but it was hardly detectable in DU145 and PC3 cells (Figure 1a). To determine the basic role of USP14 in prostate cancer, we used the CellTiter 96 Aqueous One Solution reagent (MTS) assay to test the effect of various concentrations of IU1 (6.25, 12.5, 25, 50, 100 *μ*M), a selective and potent inhibitor of USP14, on the cell growth of LNcap cells. We found that IU1 significantly decreased the cell growth in a time- and dose-dependent manner (Figure 1b). To corroborate that the IU1-induced cell growth inhibition depends on its inhibition of USP14, we applied USP14 small interfering RNA (siRNA) or short hairpin RNA (shRNA) to knock down USP14 expression, and tested the effect of USP14 knockdown on the cell viability of LNcap cells. Similarly to IU1 treatment, USP14 knockdown significantly inhibited the growth of LNcap cells in a time-dependent manner (Figures 1c and d). Given that USP14 is expressed in both androgen-responsive and androgen-irresponsive prostate cancer cells and that both pharmacological and genetic inhibition of USP14 inhibited the proliferation of androgen-responsive LNcap cells, we next tested whether USP14 plays the same role in androgen-irresponsive prostate cancer cells. Similarly, we treated androgen-irresponsive prostate cancer DU145 and PC3 cells with various concentrations of IU1 or with USP14 siRNA, followed by the MTS assay. To our surprise, the DU145 and PC3 cells were not sensitive to the treatment of IU1 or USP14 siRNA (Figures 1g and h), suggesting that USP14 plays a more important growth-promoting role in androgen-responsive prostate cancer cells than in the androgen-irresponsive prostate cancer cells. In order to test the long-term effect of USP14 inhibition or silence on cancer cells, we measured colony formation of LNcap cells using IU1 at 50 *μ*M or stably expressing USP14 shRNA. As shown in Figures 1e and f, both USP14 inhibition with IU1 50 *μ*M and USP14 silence with specific shRNA dramatically decreased LNcap cell colony formation after 2 weeks of culture.

**Figure 2:**
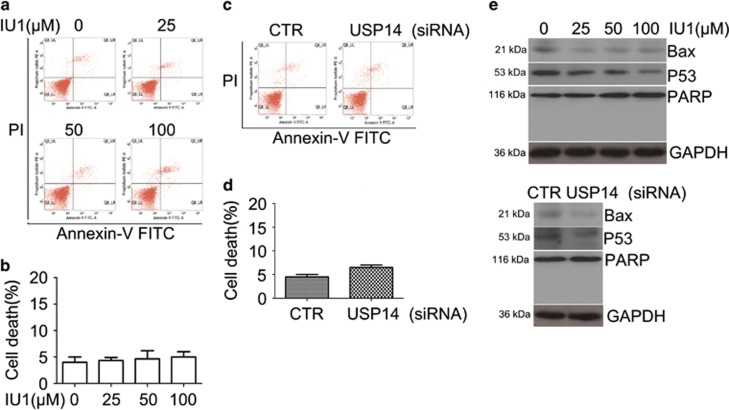
USP14 inhibition or silence failed to induce cell death in LNcap cells. LNcap cells were treated with the indicated concentrations of IU1 or USP14 siRNA for 48 h. The cultured cells were collected and stained with Annexin V-FITC/ PI, followed by flow cytometry analysis. The representative images (**a, c**) and summary of cell death (**b, d**) were shown. Mean±S.D. (*n*=3). DM, DMSO. (**e**) LNcap cells were treated with the indicated concentrations of IU1 or USP14 siRNA for 48 h. Total proteins were extracted and subjected to western blot analyses for PARP, p53 or Bax. GAPDH was used as a loading control

Since we have observed that inhibition or knockdown of USP14 inhibited cell growth in LNcap cells, we further investigated whether USP14 inhibition or silence induces cell death of LNcap cells by measuring Annexin V-FITC/PI-positive cells with flow cytometry and by measuring PARP cleavage and p53 and Bax protein expression with western blot analyses. As shown in Figures 2a−e, USP14 inhibition or silence failed to induce apoptosis or PARP cleavage but instead induced moderate decreases of p53 and Bax, suggesting that cell growth suppression mediated by USP14 inhibition or silence is through promoting cell proliferation, independent of cell death.

### USP14 promotes cell cycle by upregulating key proteins associated with the G0/G1 to S phase transition

**Figure 3:**
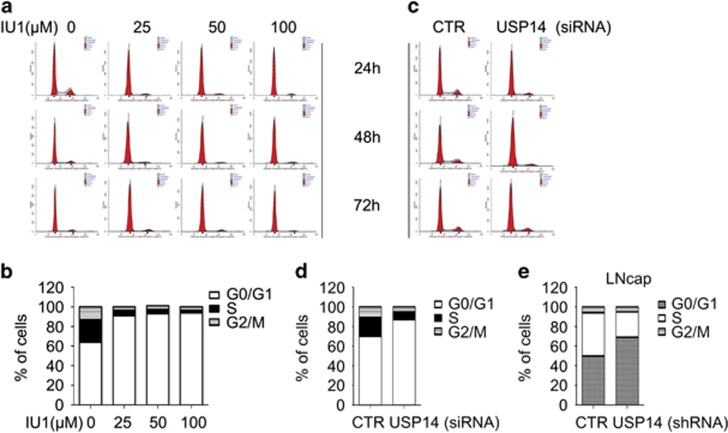
USP14 inhibition or silence induced cell cycle arrest in LNcap cells. (**a, c**) Shown are representative histograms of PI staining of LNcap. Fluorescence activated cell sorting analysis was performed on LNcap cells that stably expressed USP14 shRNA or control shRNA, or were exposed to the indicated concentrations of IU1 or USP14 siRNA for 24, 48 and 72 h. Three independent experiments were performed. The percentage of cells in each population in each cell cycle phase at 48 h was calculated (**b, d, e**)

**Figure 4:**
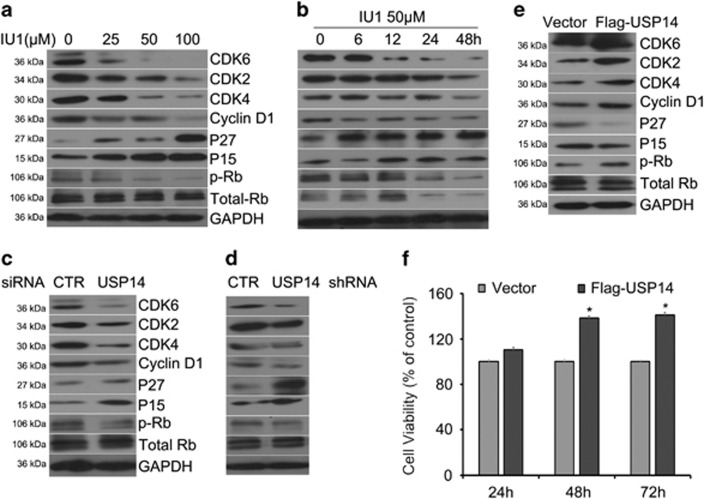
USP14 regulates G1-S transition. (**a**−**e**) Representative images of western blot analyses for key proteins associated with G1-S phase transition. Total proteins were extracted from LNcap cells that had been manipulated as described below, and subjected to western blot analyses for CDK6, CDK4, CDK2, cyclinD1, p27, p15, phospho-Rb (p-Rb) and Rb. The LNcap cells were treated with the indicated concentrations of IU1 (**a**), with 50 *μ*M IU1 for the indicated durations (**b**), with transfection of USP14 siRNA or control siRNA for 48 h (**c**), with stable expression of USP14 shRNA or control shRNA (**d**), or with transfection with Flag-USP14 plasmids or control vectors (**e**). GAPDH was used as a loading control. Three independent repeats were performed for each experiment. (**f**) MTS assay for LNcap cells transfected with Flag-USP14 plasmids or control vectors for 24, 48 and 72 h. Error bars correspond to 95% confidence intervals of three independent experiments. *P<0.05 using two-sided *t*-test

To explore the underlying mechanism by which USP14 promotes cell proliferation in LNcap cells, we monitored the cell cycle progression of each group exposed to various concentrations of IU1 (25, 50, 100 *μ*M) and found that inhibition of USP14 activity dramatically induced G0/G1 cell cycle arrest at different time points (0, 6, 12, 24, 48 h) (Figures 3a and b). In addition, silencing USP14 expression with siRNA or stable expression of shRNA also caused G0/G1 cell cycle arrest (Figure 3c−e), indicating that USP14 promotes G1-S transition in androgen-responsive prostate cancer cells. To investigate the molecular mechanism by which USP14 promotes cell cycle, we performed western blot to detect several key proteins that are associated with G1-S phase transition. We found that USP14 inhibitor IU1 decreased the expression of cyclin D1, CDK4, CDK6 and CDK2, as well as the phosphorylation/inactivation of Rb, which drive cell cycle progression from G1 to S phase, and increased the expression of p27 and p15, which block G1-S phase transition (Figures 4a and b). To further verify whether USP14 regulates these proteins in a stable status, we silenced USP14 expression with siRNA or stably expressing USP14 shRNA in LNcap cells and found that the expression of cyclin D1, CDK4, CDK6, CDK2 and phosphorylation/inactivation of Rb were decreased (Figures 4c and d), while the expression of p27 and p15 were increased as a result of USP14 knockdown. Conversely, overexpression of USP14 induced increases in the protein level of cyclinD1and CDK6/4/2, the inactivation of Rb, and decreases in the expression of p27 and p15 (Figure 4e), and led to increased proliferation of LNcap cells (Figure 4f). Taken together, these results indicate that USP14 promotes G1-S phase transition by upregulating cyclin D1, CDK4, CDK6 and CDK2 and downregulating p27 and p15 expression.

### USP14 inhibition downregulates AR and PSA and increases MDM2 expression

**Figure 5:**
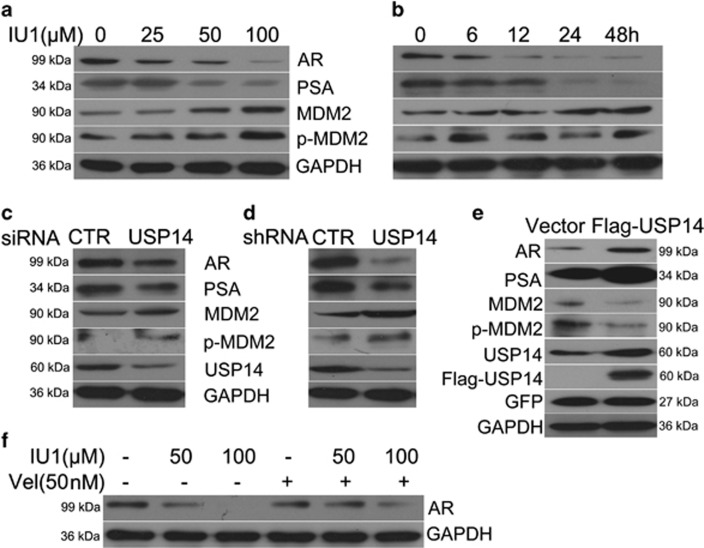
USP14 inhibits the expression and phosphorylation of MDM2, stabilizes AR, and increases PSA level. (**a-d**) Representative images of western blot analyses for AR, PSA, MDM2 and Ser166-phosphorylated MDM2 (p-MDM2) in the total proteins extracted from LNcap cells that were treated with the indicated concentrations of IU1 (**a**), IU1 50 *μ*M for the indicated duration (**b**), or transfection of USP14 siRNA or control siRNA (CTR) (**c**), or from LNcap cells stably expressing USP14 shRNA or control shRNA (**d**). GAPDH was used as a loading control. Three independent experiments were performed. (**e**) LNcap cells stably expressing Flag-USP14 or control vector were harvested. Total proteins were extracted and subjected to western blot analyses for AR, PSA, MDM2 and p-MDM2. GAPDH was used as a loading control. Flag and GFP were used as indicators of transfection efficiency. (**f**) LNcap cells were exposed to the indicated concentrations of IU1 in the absence or presence of with proteasome inhibitor bortezomib (Vel, 50 nM) for 48 h. Total proteins were extracted and subjected to western blot analyses for AR

We then sought to address why USP14, which is expressed in both androgen-responsive and -irresponsive prostate cancer cells, plays a starkly different role between the two types of prostate cancer cells. Generally, AR is over-expressed and the AR signalling pathway is extraordinarily activated in androgen-responsive prostate cancer cells; by contrast, the expression of AR is extremely low in androgen-irresponsive prostate cancer cells and the growth and proliferation of these cells is independent of AR. Therefore, we hypothesized that USP14 inhibition-induced cell cycle arrest is dependent on the expression and responsiveness of AR. To test this hypothesis, we examined the AR protein level in LNcap cells exposed to various concentrations of IU1 for different durations and found that IU1 decreased the protein levels of AR and PSA (a target gene of PSA) in a time- and dose-dependent manner, and markedly increased the expression and phosphorylation of MDM2, which is one of the known E3 ligases of AR that promotes AR degradation (Figures 5a and b). Meanwhile, a moderate decrease in p53 was observed (Figure 2e), which might be attributable to the increase of MDM2 because MDM2 promotes p53 degradation by ubiquitylating p53. Silencing the expression of USP14 with siRNA or shRNA likewise led to the same results as IU1 treatment (Figures 5c and d). Conversely, overexpression of USP14 increased the expression of AR and PSA and decreased the expression of MDM2 and its phosphorylation (Figure 5e). Moreover, IU1-induced decreases of AR were rescued by bortezomib (50 nM), a specific proteasome inhibitor (Figure 5f), suggesting that AR is degraded by the 26S proteasome, which is in accordance with previous reports, and that IU1 promotes AR degradation by the proteasome to a great extent.

### USP14 inhibits the ubiquitination and degradation of AR

**Figure 6:**
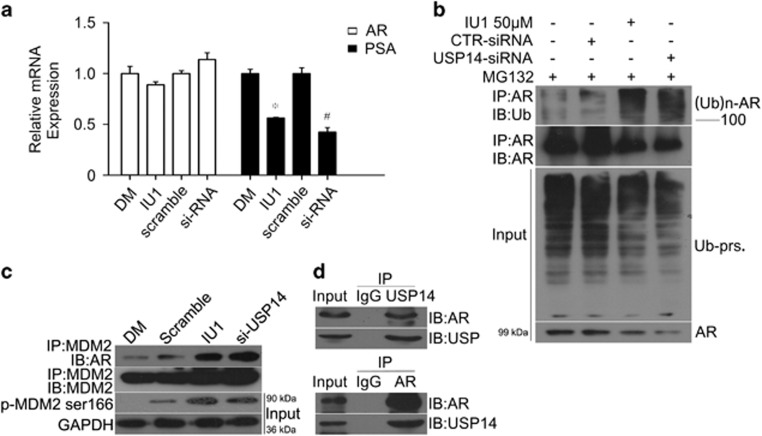
USP14 reduces the ubiquitination of AR and stabilizes AR proteins. (**a**) LNcap cells were exposed to IU1 50 *μ*M or USP14 siRNA for 24 h. Total RNAs were extracted and subjected to RT^2^-PCR analysis. GAPDH was used as an internal control. Three independent experiments were performed. Mean±S.D. (*n*=3). *P<0.05, compared with DM. ^#^*P*<0.01, compared with scramble siRNA. (**b**) LNcap cells were exposed to IU1 50 *μ*M or USP14 siRNA for 48 h, immunoprecipitated with AR antibody beads and immunoblotted for ubiquitin (Ub) and AR. Cells were treated with MG132 (10 *μ*M) for 6 h before harvest. (**c**) LNcap cells were exposed to IU1 50 *μ*M or USP14 siRNA for 48 h, immunoprecipitated with MDM2 antibody beads and immunoblotted for AR and MDM2. (**d**) Total proteins were extracted from LNcap cells, immunoprecipitated with USP14 antibody beads and immunoblotted for AR and USP14 (upper), and immunoprecipitated with AR antibody beads and immunoblotted for AR and USP14 (lower)

As shown above, USP14 decreases the expression and phosphorylation of MDM2. We further detected the change of MDM2-AR interaction using co-immunoprecipitation (co-IP), and found that USP14 inhibition or silence increased the binding of MDM2 to AR (Figure 6c), suggesting that USP14 inhibits the degradation of AR by decreasing the expression and phosphorylation of MDM2. Additionally, we sought to determine that whether USP14 directly interacts with AR and serves as another DUB for AR or promotes the transcription of AR. We detected the effect of IU1 or USP14 siRNA on the mRNA expression of AR and PSA using real-time reverse transcriptase PCR (RT^2^-PCR). We found that both IU1 and USP14 siRNA dramatically decreased the PSA mRNA but not the AR mRNA, indicating that USP14 inhibition or gene silence does not affect the transcription of AR (Figure 6a). Hence, we proposed that inhibition or silence of USP14-induced AR downregulation is through enhancing the degradation of AR. To detect the interaction between AR protein and USP14 protein, we performed co-IP for USP14 and AR. We found that USP14 could directly bind AR proteins (Figure 6d). To further confirm that USP14 is a DUB of AR, we determined the effect of IU1 or USP14 siRNA on the abundance of ubiquitinated AR using co-IP. We found that IU1 and USP14 knockdown dramatically increased the ubiquitinated AR (Figure 6b), suggesting that USP14 is a DUB for AR, capable of reversing the ubiquitination of AR and thereby stabilizing AR proteins.

## Discussion

Prostate cancer remains the most frequently diagnosed non-cutaneous malignancy and a leading cause of male cancer death.^5^ The androgen deprivation therapy with gonadotropin-releasing hormone agonists or bilateral orchiectomy is the mainstay of clinical management for prostate cancer.^28^ However, the inevitable progression to castration-resistant prostate cancer, which occurs within 2−3 years after the initiation of androgen deprivation therapy, represents a major medical challenge.^29^ Additionally, patients treated with androgen deprivation therapy are at increased risk of cardiovascular events, including myocardial infarction and stroke.^30, 31^ Therefore, more therapeutic strategies as well as alternative targets are necessary for prostate cancer treatment.

A few DUBs were reported to be over-expressed in tumour tissues and some of them are emerging as a class of novel targets or biomarkers for anticancer strategy. The current study has discovered that USP14, one of the 19S proteasome-associated DUBs, is involved in the stabilization of AR proteins and promotes G0/G_1_ to S phase transition in human prostate cancer cells and also identified USP14 as a potential target for prostate cancer therapy.

Our study shows that inhibiting USP14 expression or its function leads to cell proliferation inhibition and cell cycle arrest at the G0/G1 phase in androgen-responsive prostate cancer cells but not in androgen-irresponsive prostate cancer cells. We confirmed that AR was highly expressed in the androgen-responsive prostate cancer cells (LNcap cells) but was hardly detectable in the androgen-irresponsive prostate cancer cells (DU145 and PC3 cells) tested here (Figure 1a), implying that the induction of cell cycle arrest by USP14 inhibition is AR dependent. Indeed, both pharmacological and genetic inhibition of USP14 markedly reduced, and conversely USP14 overexpression increased, the steady state protein levels of AR and its target gene PSA in LNcap cells (Figure 5). Furthermore, changes in key cell cycle regulators induced by the manipulation of USP14 function also support the notion that AR is a key target for USP14 in the prostate cancer cells. Our experiments showed that CDK4, CDK6, CDK2, cyclinD1 and phosphorylated Rb were downregulated, while p27 and p15 were increased, by inhibiting USP14 expression or its activity in androgen-responsive prostate cancer cells; and conversely, the exactly opposite changes were induced by USP14 overexpression. Recent reports have shown that AR promotes the G1-S transition in androgen-responsive prostate cancer cells by the following means.^32^ AR increases cyclin D mRNA and cyclin E activity, decreases p21 expression, and promotes p27 degradation. Typically, in the early G1 phase, cyclin Ds and CDK4 or 6 form complexes and thereby initiate phosphorylation/inactivation of the retinoblastoma tumour suppressor (Rb), a negative regulator for cell cycle transitions and the onset of DNA replication. Rb phosphorylation releases E2F and leads to G1-S transition. Protein p27 and p21 belong to the endogenous CDK inhibitors, which potently inhibit CDKs kinase activity in the G0/G1 phase and trigger cell cycle arrest.^33, 34^ The p15 protein can bind to CDK 4 and 6 and inhibit the kinase activity of the formed complex, which similarly blocks the G1-S transtion.^35^

**Figure 7:**
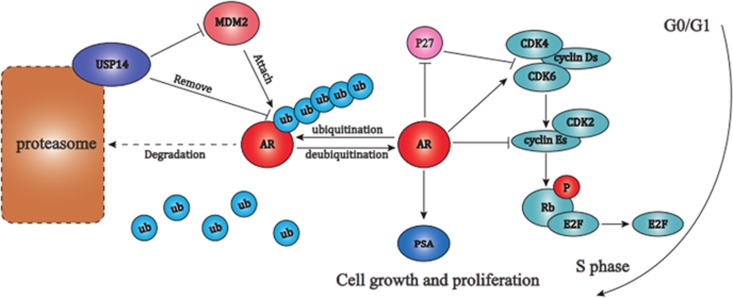
A molecular model for USP14 to regulate AR and cell cycle. MDM2 is one of the E3 ligases for AR that attaches ubiquitin (Ub) chain to AR and thereby leads to AR degradation. On one hand, USP14 indirectly decreases the ubiquitination and degradation of AR by decreasing the MDM2 protein level; on the other hand, USP14 directly deubiquitinates AR by binding AR and removing the ubiquitin chain from AR and thereby antagonizes ubiquitin proteasome system-mediated AR degradation. AR mediates cell growth and proliferation by transcriptional activation of PSA expression and promoting G1-S phase transition

The AR-dependent cell cycle arrest by inhibiting USP14 prompted us to investigate the interaction between USP14 and AR. Indeed, we found that inhibition or reduction of USP14 dramatically decreased the protein levels of AR and PSA (a target gene of AR). Our gene expression analyses of androgen-responsive prostate cancer cells exposed to IU1 or USP14 siRNA show a downregulation of PSA but not AR mRNA expression, suggesting that USP14 might not enhance the transcriptional activity of AR. In the rescue experiments, IU1-induced AR downregulation can be reversed by proteasome inhibitor bortezomib (Velcade), suggesting that reduction of AR protein levels by the inhibition of USP14 depends on proteasome activity. Thus we present a hypothesis that USP14 increases AR by inhibiting AR degradation, not by promoting AR transcription. Furthermore, we found that the binding of AR to MDM2, as well as the levels of native and phosphorylated MDM2, a known E3 ligase of AR, which assists AR ubiquitination and degradation in physiological condition, were significantly increased upon inhibiting USP14 activity or expression. The increased MDM2 can also promote AR ubiquitination and degradation. More importantly, we observed that USP14 could bind to AR and decreased AR ubiquitination, suggesting that USP14 may be another DUB of AR (Figure 7).

In summary, via demonstrating the role of USP14 in AR stabilization in androgen-responsive prostate cancer, here we provide a potentially new strategy for inhibiting AR-mediated prostate cancer carcinogenesis or progression through inhibition of USP14.

## Materials and methods

### Materials

IU1, siRNA and shRNA were obtained from Santa Cruz Biotechnology (Santa Cruz, CA, USA). MG132 and bortezomib (Velcade) were purchased from Selleckchem (Houston, TX, USA). MTS assay (CellTiter 96 Aqueous One Solution reagent) was purchased from Promega Corporation (Madison, WI, USA). Propidium iodide (PI) and Annexin V-FITC Apoptosis Detection Kit were purchased from Keygen Company (Nanjing, China). Dynabeads antibody coupling kit was from Life technologies. Antibodies used in this study were purchased from following sources: anti-ubiquitin (P4D1) (Santa Cruz Biotechnology); anti-PARP, anti-CDK2, anti-phospho-Rb, anti-Rb, anti-PSA, anti-Bax, anti-GFP, anti-GAPDH (Bioworld Technology, Inc., Louis Park, MN, USA); anti-CDK4, anti-CDK6, anti-phospho-MDM2, anti-P53, anti-USP14, anti-Flag, anti-cyclin D1, anti-p15, anti-p27 (Cell Signaling Technology, Beverly, MA, USA); anti-MDM2 and anti-AR (Abcam, USA).

### Cell lines and cell culture

Human prostate cell lines LNcap, PC3 and DU145 were purchased from American Type Culture Collection (Manassas, VA, USA). LNcap grown in RPMI 1640 supplemented with 10% FBS. PC3 and DU145 grown in Hyclone DMEM/F-12 supplemented with 10% FBS. Cultured cells were maintained at 37 °C and 5% CO_2_.

### Cell viability assay

MTS assay (CellTiter 96 Aqueous One Solution reagent) was used to test cell viability as we previously reported.^36^ In brief, exponentially growing LNcap, PC3 or DU145 cells were seeded at 2500 cells/well in a 96-well plate. After incubation for 24 h, cells were treated with IU1, usp14 siRNA or shRNA, followed by continuous incubation for 24, 48 or 72 h. 20 μl MTS reagent was directly added to each well and the incubation was continued for additional 3 h. The absorbance of optical density was measured with a microplate reader (Sunrise, Tecan, Mannedorf, Switzerland) at wavelength 490 nm. Cell viability was calculated by the following formula: cell viability (%)=(average absorbance of treated group−average absorbance of blank)/(average absorbance of untreated group−average absorbance of blank) × 100%.

### Cell cycle and cell death assay

For cell cycle assay, LNcap cells were harvested and washed with 4 °C PBS twice and the precipitated cells were resuspended with 2 ml 70% ethanol at 4 °C overnight. And then the cells washed with 4 °C PBS twice again, followed by incubation with PI (50 μg/ml), RNase A (100 μg/ml) and 0.2% Triton X-100 complexes for 30 min at 4 °C in dark. The stained cells were analysed with flow cytometry. Apoptosis assay was performed as previously described.^37^ Briefly, cultured LNcap cells were harvested and washed with 4 °C PBS twice and resuspended with the binding buffer, followed by Annexin V-FITC incubation for 15 min and PI staining for another 15 min in dark. The stained cells were analysed with flow cytometry within 30 min.

### Clonogenic assay

This assay was performed as we previously described.^38^ LNcap cells exposed to either IU1 or USP14 shRNA were suspended in 60-mm dishes carrying 30% agarose supplemented with 10% FBS RPMI-1640 medium then cultured in an atmosphere of 5% CO2 for 10 days, then stained with 0.3% crystal violet solution. The colonies >60 *μ*M in diameter were counted under the light microscope. The experiments were done in three independent repeats.

### SiRNA and shRNA transfection

This assay was performed as we previously described.^39^ To knock down USP14 expression in prostate cancer cells, siRNA or shRNA targeting human USP14 were synthesized and purchased from Santa Cruz Biotechnology Inc.. siRNA or shRNA with non-specific sequences were used as control scrambled RNA. Different siRNAs and shRNAs were transfected separately into cells using Lipofecatmine 2000 (Invitrogen, Carlsbad, CA, USA) reagent and the medium was replaced 6 h after the transfection.

### Lentivirus overexpressing USP14 transfection

Lentivirus (pLent-EF1a-FH-CMV-GP) overexpressing Flag-USP14 (NM-005151) or containing control vector was purchased from VigeneBio (Shandong, China). Exponentially growing LNcap cells were seeded in six-well plates. After culture overnight and reaching 50% confluence, medium containing lentiviruses and polybrene (5 μg/ml; Santa Cruz) was added at a multiplicity of infection of 10 and mixed with the cells. After incubation overnight, the supernatant at each well was replaced with RPMI 1640 containing 10% FBS and cultured for 48 h. For selection of stably transfected cells, we proceeded with puromycin selection as follows: replacing the medium with fresh medium containing puromycin (Santa Cruz) at the concentration of 1 μg/ml. Every 2 days, the medium was replaced with freshly prepared selective medium to culture the survived cells.

### RNA extraction and real-time reverse transcriptase polymerase chain reaction (RT^2^-PCR) analysis

Total RNA was isolated from the pooled cells using an RNeasy Mini Kit (Qiagen, Hilden, Germany) according to the manufacturer's instructions. The total RNA was digested by DNase I (TaKaRa Biotechnology, Dalian, China) to remove genomic DNA contamination. The purified total RNA was measured at 260 and 280 nm using a Bio-Rad SmartSpec 3000 spectrophotometer (Bio-Rad, CA, USA). The 260:280 nm ratios and a 1% agarose-formaldehyde gel stained with ethidium bromide were used to verify the quality of the RNA in each sample. The first-strand cDNA was synthesized from 1 μg total RNA using a PrimeScript II 1st Strand cDNA Synthesis Kit (TaKaRa Biotechnology). The expression levels of target genes were detected by real-time quantitative PCR, with the housekeeping gene GAPDH being used as an internal control. SYBR Green PCR Master Mix reagent kits (Applied Biosystems Inc, USA) were used according to the manufacturer's instructions for quantification of gene expression with a 7500 real-time PCR system (Applied Biosystems Inc.). PCR primers are as following, AR forward: 5′-GGTGAGCAGAGTGCCCTATC-3′, AR reverse: 5′-GAAGACCTTGCAGCTTCCAC-3′ PSA forward: 5′-AGGCCTTCCCTGTACACCAA-3′, PSA reverse: 5′-GTCTTGGCCTGGTCATTTCC-3′ GAPDH forward: 5′-TCCCATCACCATCTTCCA-3′, GAPDH reverse: 5′-CATCACGCCACAGTTTCC-3′. The cycling conditions were as follows: 95 °C for 10 s followed by 40 cycles of 95 °C for 15 s and 60 °C for 1 min. After PCR, a melting curve analysis was performed to demonstrate the PCR product specificity. Every sample was analysed in triplicate. The relative expression level of a target gene was presented as the sample versus the control.

### Western blot and co-IP analyses

For IP and western blot, dynabeads m-270 Epoxy (Invitrogen) coupled with antibodies were prepared and then cell lysates were added, and the antibodies−lysate mixtures were rotated at 4 °C for 1 h. Immunocomplexes separated from the dynabeads were washed with lysis buffer and then suspended with SDS blue loading buffer. To detect ubiquitinated proteins, lysis was performed under 80 °C for 10 min. Western blot analysis was performed as we described previously.^40^ In brief, an equal amount of total protein extracted from cultured cells were separated by 12% SDS–PAGE and transferred to polyvinylidene difluoride membranes. The blots were blocked with 5% milk for 1 h. Primary Abs and horseradish peroxidase-conjugated secondary Abs were each incubated for 1 h. The bounded secondary antibodies were reacted to the ECL detection reagents and exposed to X-ray films (Kodak, Japan).

### Statistical methods

Mean±S.D. are presented where applicable. Unpaired Student's *t*-test or one-way ANOVA is used where appropriate for determining statistic probabilities. GraphPad Prism5.0 software (GraphPad Software) was used for statistical analysis. *P* value less than 0.05 was considered statistically significant.
